# Comparative multi‐omics in female mice reveals tissue‐specific vulnerabilities to chronic alcohol intake

**DOI:** 10.1111/acer.70240

**Published:** 2026-01-30

**Authors:** Craig R. G. Willis, Muni Swamy Ganjayi, Austin M. Brown, Samantha E. Moser, Nathaniel J. Szewczyk, Brian C. Clark, Cory W. Baumann

**Affiliations:** ^1^ Research Software and Analytics University of Exeter Exeter UK; ^2^ Department of Biomedical Sciences, Heritage College of Osteopathic Medicine Ohio University Athens Ohio USA; ^3^ Ohio Musculoskeletal and Neurological Institute Ohio University Athens Ohio USA; ^4^ Honors Tutorial College Ohio University Athens Ohio USA; ^5^ Institute for Molecular Medicine and Aging Ohio University Athens Ohio USA

**Keywords:** alcohol, lipidomics, liver, metabolomics, multi‐omics, proteomics, skeletal muscle, transcriptomics

## Abstract

**Background:**

Alcohol‐related liver disease (ALD) and alcohol‐related myopathy are consequences of chronic alcohol use. However, understanding of the associated molecular mechanisms and effective treatments remains limited.

**Methods:**

Multi‐omics were employed to uncover molecular blueprints of liver versus skeletal muscle responses to chronic alcohol exposure, using a preclinical mouse model showing signs of alcohol‐related liver dysregulation (diminished liver phosphatidylcholine‐to‐phosphatidylethanolamine lipid ratio) and alcohol‐related myopathy (reduced muscle mass and strength).

**Results:**

A greater proportion of transcriptomic, proteomic, and metabolomic features were altered by alcohol in liver than muscle, whereas similar proportions of lipid species were affected in both tissues. The liver was significantly enriched for a broad and diverse set of metabolic pathways across molecular layers, while muscle was associated with upregulated inflammatory and matrisome responses and impaired mitochondrial energetics. Lipidome analyses also revealed a novel potential role for altered phospholipid remodeling in the etiology of alcohol‐related myopathy. Finally, computational drug repurposing identified several compounds for therapeutic targeting of alcohol‐induced liver (e.g., saracatinib, GSK126) and muscle (e.g., metformin, trichostatin A) pathophysiology.

**Conclusions:**

Overall, this study provides a list of therapeutic targets and treatments to help expedite the understanding of and countermeasures against ALD and myopathy in humans.

## Graphical abstract

Using mice, we applied multi‐omics to compare liver and skeletal muscle after chronic alcohol consumption. The liver responded more strongly across genes, proteins, and metabolites, with a lowered PC:PE ratio. Muscle showed inflammation, extracellular matrix activation, impaired mitochondrial energetics, and an elevated PC:PE ratio. Lipidomics affected both tissues and pointed to altered phospholipid remodeling in myopathy. Drug repurposing suggested liver candidates (saracatinib, GSK126) and muscle candidates (metformin, trichostatin A). This work outlines tissue‐specific mechanisms and practical targets/therapies for alcohol‐related disease.

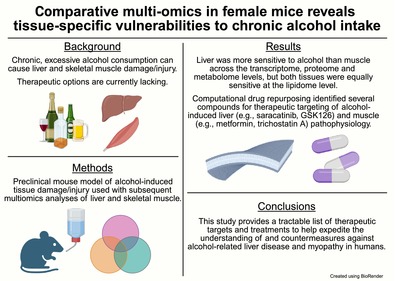

## INTRODUCTION

Alcohol use remains prevalent across society. Chronic, heavy alcohol consumption is well known to cause numerous adverse health outcomes affecting multiple organ systems (Rehm et al., 2010). Because the liver is the primary site of alcohol metabolism, it is among the organs most severely impacted by prolonged alcohol intake (Hyun et al., 2021). Excessive alcohol use can lead to alcohol‐related liver disease (ALD), which encompasses a broad clinical spectrum, including alcohol‐related fatty liver (steatosis), alcohol‐related hepatitis, cirrhosis, and liver cancer (Osna et al., 2017). Alcohol‐related fatty liver occurs in up to 90% of heavy alcohol drinkers and, while reversible with abstinence, can progress to more severe liver disease (e.g., cirrhosis) and increase the risk of liver‐related mortality (Haflidadottir et al., 2014; Toshikuni et al., 2014). Liver cirrhosis is the 11th leading cause of death worldwide and develops in 10%–20% of heavy alcohol users, with 30%–50% of global cirrhosis‐related deaths attributable to alcohol (Cheemerla & Balakrishnan, 2021; Hernandez‐Evole et al., 2024). Despite ALD being the most prevalent type of chronic liver disease globally (Rehm & Shield, 2019), therapeutic options are still lacking. Abstinence can be challenging for alcohol‐dependent individuals and cannot reverse advanced stages of ALD (Becker, 2008; Singal & Anand, 2013). Nevertheless, there are currently no approved pharmacologic treatments for ALD.

Another major organ impacted by alcohol is skeletal muscle. Excessive alcohol intake can cause muscle atrophy and weakness, a condition known as alcohol‐related myopathy (Urbano‐Marquez & Fernandez‐Sola, 2004). Skeletal muscle is central to many key physiological processes, including locomotion and metabolism. Alcohol‐induced muscle atrophy can thus have profound negative implications on physical function and overall health, reducing quality of life (Simon et al., 2017) and increasing mortality risk (Shibamoto et al., 2025). Strength loss can exceed 30% in chronic, heavy alcohol drinkers and is only partially restored with abstinence (Estruch et al., 1998). With alcohol‐related myopathy affecting 45%–70% of heavy drinkers, it is one of the most widespread alcohol‐related conditions (Souza‐Smith et al., 2016). The exact pathophysiologic mechanisms of alcohol‐related myopathy remain incompletely defined and likely multifactorial, with impaired muscle protein synthesis, increased muscle protein degradation, mitochondrial dysfunction, perturbed metabolism, inflammation, oxidative stress, and diminished muscle regenerative capacity all purported to contribute (Simon et al., 2017). Moreover, like ALD, therapeutic strategies for alcohol‐related myopathy are limited.

The development and progression of ALD and alcohol‐related myopathy are likely caused not only by the direct effects of alcohol on liver and muscle, but also via indirect effects across these two tissues. Indeed, ALD may contribute to alcohol‐related myopathy, and vice versa, by influencing signaling between the liver and skeletal muscle (Dasarathy et al., 2017; Shibamoto et al., 2025; Simon et al., 2017). Certain alcohol‐induced phenotypes are common between the liver and muscle, such as tissue fibrosis (Dekeyser et al., 2013; Osna et al., 2017). Thus, ALD and alcohol‐related myopathy encompass distinct, shared, and interlinked pathophysiologic hallmarks. Understanding similarities and differences in molecular responses of liver and skeletal muscle to chronic alcohol exposure could therefore expedite the development of more targeted therapeutic interventions for both conditions. However, the molecular dynamics of excessive alcohol consumption in the context of liver versus skeletal muscle remain largely unknown.

Molecular and translational medicine have been revolutionized by the paradigm shift of biology into a “big data” era. Indeed, the integration and interrogation of different omics data types, termed “multi‐omics,” can improve biological insights by providing a more holistic “systems‐level” view of disease etiology. In turn, multi‐omics offers an unparalleled platform for aiding the discovery and development of new disease diagnostics, prognostics, treatments, and preventative strategies. Therefore, we harnessed the power of multi‐omics to define and compare the molecular blueprints of chronic alcohol consumption in liver and skeletal muscle using an established preclinical mouse model. Our findings provide valuable insights into the molecular mechanisms underlying alcohol‐induced liver and skeletal muscle pathophysiology and reveal new therapeutic targets and candidate drug options. Consequently, the results from this innovative multi‐omics study provide a strong foundation for accelerating the understanding of, and targeted treatments against, ALD and alcohol‐related myopathy in humans.

## MATERIALS AND METHODS

### Experimental overview

The mice and experimental procedures were conducted as previously described (Moser et al., 2022, 2023). Briefly, female C57BL/6 mice obtained from Jackson Laboratory were aged 23–28 weeks and randomly assigned access to either a 100% water bottle (Control mice; *n* = 9) or a bottle containing 80% water + 20% alcohol (ethanol) (Alcohol mice; *n* = 14) for 34–40 weeks. Female mice were selected because previous studies have shown that females are more susceptible to alcohol‐related myopathy and ALD (Becker et al., 1996; Shenkman et al., 2016; Urbano‐Marquez et al., 1995). Alcohol‐consuming mice were gradually acclimated to alcohol by increasing the concentration in 5% increments (from 0% to 20% w/v) over a 2‐week period. All mice had continuous access to standard rodent chow ad libitum throughout the study. At study completion, body composition was assessed using the Bruker Minispec NMR Analyzer (LF50 Series, model mq 7.5), in vivo plantarflexor muscle isometric torque of the left leg using a servomotor system (Model 300C‐LR and 701C; Aurora Scientific, Aurora, ON, Canada), and blood alcohol concentration using an AM1 alcohol analyser (Analox Instruments Ltd.). Mice were euthanized under anesthesia (2%–3% isoflurane) by exsanguination followed by cervical dislocation, and then, the posterior crural muscles (gastrocnemius, soleus, and plantaris) of the left leg and liver were harvested, weighed, snap‐frozen in liquid nitrogen, and stored at −80°C until further analyses. For the purposes of this work, a composite of blood alcohol concentration, body composition, and muscle mass and torque data from two previous publications has been included (Moser et al., 2022, 2023). Experimental procedures were approved by the Ohio University Animal Care and Use Committee. All methods were performed in accordance with the relevant guidelines and regulations.

### Omics data generation

Liver and muscle tissues were harvested and shipped on dry ice to BGI Americas (San Jose), where samples were processed and omics data were generated. For each omics‐based analysis, approximately 55–95 mg of liver tissue and 35–60 mg of muscle tissue were used. Because the entire posterior crural muscle compartment was dissected and snap‐frozen as a single unit, muscle subsampling was performed with care to ensure the same anatomical portion was used across analyses, given the compartment's heterogeneity in muscle groups and fiber types.

For transcriptomics, strand‐specific (second strand cDNA synthesis with dUTP) 100 bp paired‐end reads were generated from extracted RNA using the DNBseq platform (further experimental details are provided as part of the metadata for the raw data's entry in the NCBI Sequence Read Archive repository; see Data Availability Statement herein). For proteomics, a label‐free quantitative approach was undertaken using nano flow HPLC (Ultimate 3000) followed by Orbitrap Eclipse Tribrid Mass Spectrometer (Thermo Fisher Scientific, USA). A spectral library was constructed via data‐dependent acquisition using the MS2‐based method, using a fractionated composite of all digested samples. Data‐independent acquisition was subsequently performed on each sample via a high‐resolution full mass spectrometry scan followed by two data‐independent acquisition segments. Raw mass spectrometer files were input into MaxQuant (v1.5.3.30; https://www.maxquant.org/) for identification and quantification against the mouse database, with identified peptides that satisfied a false discovery rate ≤1% used when constructing the final spectral library. For metabolomics and lipidomics, features were extracted from tissue samples using solvent‐based precipitation, with feature separation and detection performed using a UPLC I‐Class Plus (Waters, USA) tandem Q Exactive high‐resolution mass spectrometer (Thermo Fisher Scientific), utilizing AQUITY UPLC BEH C18 and Amide columns for metabolites and a CSH C18 column for lipids (Waters). In any case, QC samples were produced by pooling equal volumes of prepared supernatant (10 *μ*L) from each sample. Off‐line mass spectrometry data were subsequently input into Compound Discover (v3.3; Thermo Fisher Scientific) for metabolite peak extraction and identification (using BGI metabolome, mzcloud and chemspider databases as reference) or LipidSearch (v4.1; Thermo Fisher Scientific) for lipid peak extraction and identification (further experimental details are provided as part of the raw data's entry in the EBI MetaboLights repository; see Data Availability Statement herein).

### Omics data preprocessing

For transcriptomic data, reads were cleaned using SOAPnuke software (v2.2.1; https://github.com/BGI‐flexlab/SOAPnuke) to filter out adaptor sequences, contamination, and low‐quality reads. The clean reads were then aligned to the mouse reference transcriptome using Kallisto (v0.46.1, with bias correction; https://pachterlab.github.io/kallisto/). The reference transcriptome was compiled from the Ensembl Release 109 mouse reference genome (primary assembly) and associated transcript annotations using GFFread (v0.12.7; https://github.com/gpertea/gffread). Gene counts were inferred from transcript abundance estimates scaled to library size using the tximport R package (v1.28.0; https://doi.org/10.18129/B9.bioc.tximport). Lowly expressed genes were then removed using the edgeR R package (v3.42.4; https://doi.org/10.18129/B9.bioc.edgeR) *filterByExpr* function (with tissue as the grouping factor, “min.prop” set to 1, and “min.count” kept as its default value of 10), and counts for remaining genes (*n* = 14,017) normalized using the limma‐voom approach as per developer guidelines for mixed‐design studies. For proteomic data, log_2_ transformation was applied, proteins with more than 50% missing values omitted and the *k*‐Nearest Neighbour Algorithm used to impute missing values, resulting in normalized abundances for *n* = 3512 proteins. For metabolomics data, each UPLC column's Compound Discover output was subject to probabilistic quotient normalization to obtain relative peak areas, quality control‐based robust LOESS signal correction to correct batch effect, removal of features with a coefficient of variation larger than 30% based on relative peak area in QC samples, filtering for features with a recognized KEGG ID, and log_2_ transformation. Normalized metabolite abundances for each column were then aggregated based on mean value to produce a singular metabolomic dataset (*n* = 873 features). For lipidomic data, the LipidSearch output was subject to removal of lipids with >50% missing values in QC samples and >80% missing values in experimental samples, imputation of remaining empty values via the *k*‐Nearest Neighbour Algorithm, probabilistic quotient normalization to obtain relative peak areas, quality control‐based robust LOESS signal correction to correct batch effect, filtering to remove features with a coefficient of variation larger than 30% based on relative peak area in QC samples, and log_2_ transformation, leading to normalized abundances of *n* = 744 lipids. Prior to log_2_ transformation, percent content of PC and PE lipid classes in each sample (sum of relative peak values for a given class in the sample, divided by total of all relative peak values in the sample) were also calculated and used to quantify muscle and liver PC:PE ratios.

### Statistics

#### Statistical evaluation of end‐point data

End‐point variables (total body mass, lean mass, fat mass, plantarflexor muscle mass, plantarflexor muscle torque, liver mass, liver‐to‐body mass ratio, plantarflexor muscle PC:PE ratio, liver PC:PE ratio, and blood alcohol concentrations) were compared between alcohol‐consuming mice and control mice on a per‐tissue basis using either the two‐tailed Student's independent *t*‐test (when normal distribution and equal variance), the two‐tailed Welch's independent *t*‐test (when normal distribution but unequal variance), or the Mann–Whitney *U*‐test (when non‐normal distribution). Analyses were conducted in R (v4.3.1; https://www.r‐project.org/), with statistical significance accepted when *p* ≤ 0.05. Unless otherwise stated, in‐text descriptive statistics are presented as median (interquartile range). See Appendix S1 for omics‐based statistical analyses.

## RESULTS

### Mice that drink alcohol chronically show signs of alcohol‐related myopathy and ALD

**FIGURE 1:**
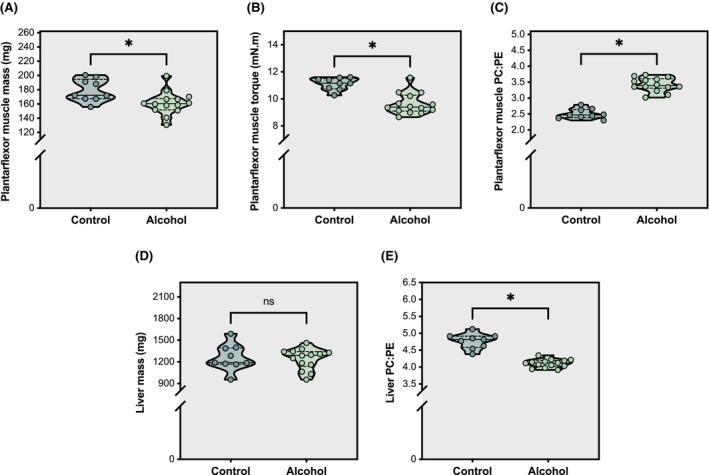
Indicators of alcohol‐related myopathy and alcohol‐related liver disease (ALD) following chronic alcohol consumption. Violin plots illustrate plantarflexor muscle mass (Panel A), plantarflexor muscle peak isometric contractile torque (Panel B), plantarflexor muscle PC:PE ratio (Panel C), liver mass (Panel D), and liver PC:PE ratio (Panel E) in alcohol‐consuming mice versus control mice. Solid lines within violin shapes indicate median, while dashed lines illustrate quartiles. Analyses via two‐tailed Student's/Welch's independent *t*‐test or Mann–Whitney *U*‐test, as appropriate. *p ≤ 0.05.

Transcriptomic, proteomic, metabolomic and lipidomic data were generated from the liver and the left plantarflexor muscles (i.e., posterior crural muscle: gastrocnemius, plantaris, soleus) of adult C57BL/6 female mice that had consumed either 100% water (Control mice, *n* = 9) or 80% water + 20% alcohol (ethanol) (Alcohol mice, *n* = 14) for 34–40 weeks (Moser et al., 2022, 2023). Mice that consumed alcohol had elevated blood alcohol levels (Control = 0 (3.1) mg dL^−1^, Alcohol = 159.3 (120.6) mg dL^−1^; *p* = 7.60E‐05) and lower total body mass (Control = 28.1 (6.5) g, Alcohol = 25.8 (3.7) g; *p* = 5.55E‐03), fat mass (Control = 7.0 (2.1) g, Alcohol = 5.4 (1.9) g; *p* = 1.73E‐02) and lean mass (Control = 18.5 (2.5) g, Alcohol = 17.0 (1.1) g; *p* = 1.34E‐03) compared with controls. Plantarflexor muscle mass (Figure 1A) and in vivo isometric torque (Figure 1B) were also lower in alcohol‐consuming mice relative to control mice, consistent with alcohol‐induced muscle atrophy and weakness (i.e., alcohol‐related myopathy).

Phosphatidylcholine (PC) and phosphatidylethanolamine (PE) are the most abundant membrane phospholipids, and the PC:PE ratio governs membrane biophysics; higher ratios increase stability and rigidity, whereas lower ratios increase curvature, fluidity, and fragility. In skeletal muscle, we observed an elevated PC:PE ratio (Figure 1C), a lipid signature associated with insulin resistance (e.g., type 2 diabetes) and reversed by exercise (Lee et al., 2018; Newsom et al., 2016). This shift indicates a metabolically rigid, insulin‐resistant state characteristic of alcohol‐related myopathy and is predicted to impair the membrane remodeling required for insulin‐stimulated GLUT4 translocation and proper Ca^2+^ handling. Although liver mass was similar between control and alcohol‐consuming mice (Figure 1D), chronic alcohol intake lowered the hepatic PC:PE ratio (Figure 1E). A reduced PC:PE ratio promotes excessive curvature and fluidity, compromising plasma, mitochondrial, and endoplasmic reticulum membranes and predisposing to mitochondrial permeability transition, ER stress, and necrosis—features of alcohol‐induced hepatitis (Li et al., 2006), and is consistent with diminished hepatic membrane potential and indicators of ALD in preclinical models and patients (Jeon & Carr, 2020).

### Chronic alcohol consumption extensively disrupts liver and muscle transcriptomes

**FIGURE 2:**
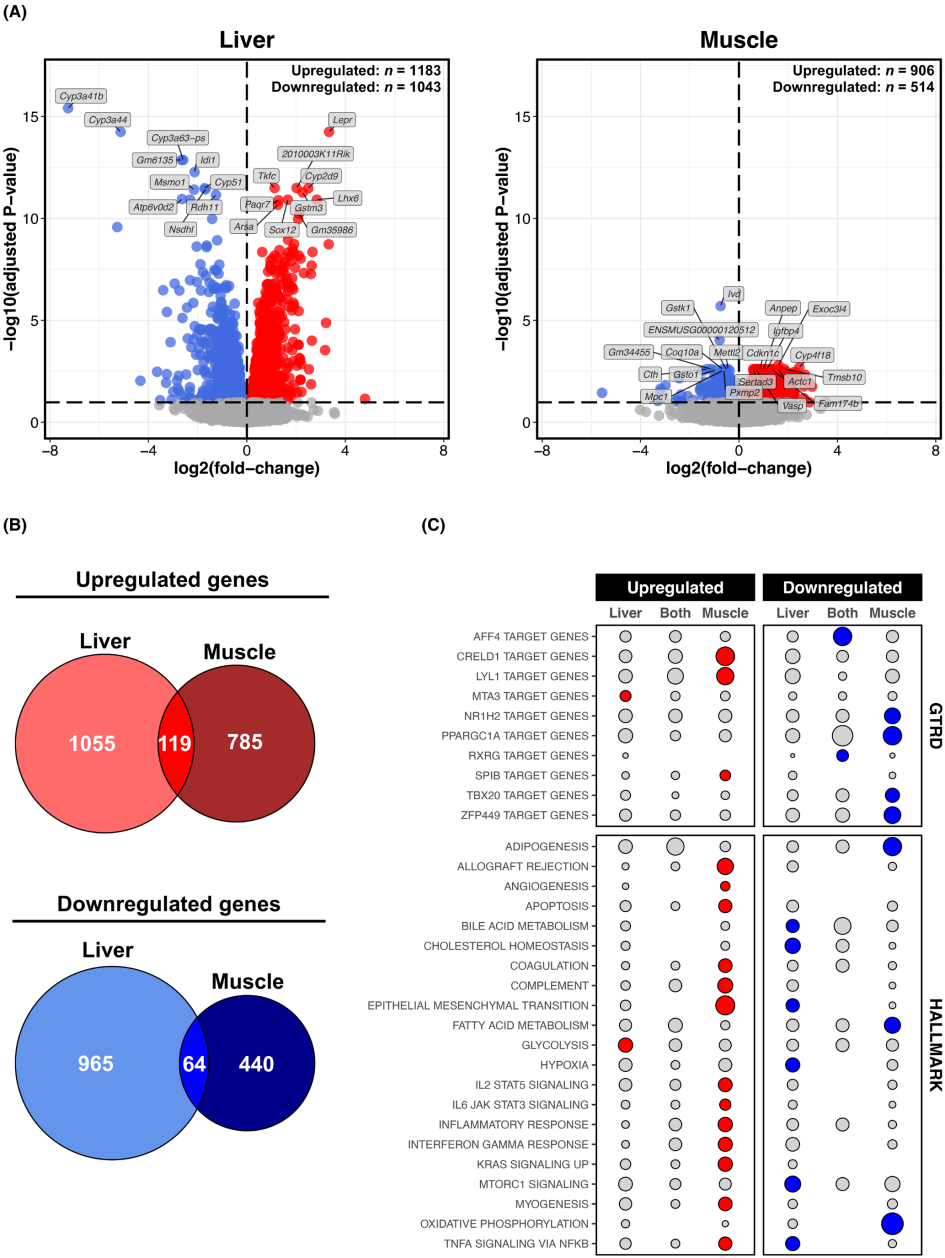
Liver versus muscle transcriptomic responses to chronic alcohol. (Panel A) Volcano plots for differential gene expression analysis (Alcohol vs. Control) in the liver and muscle. Red and blue shading denote significant upregulation and downregulation, respectively (adjusted *p* ≤ 0.1). Annotated genes are those ranked in top 10 upregulated/downregulated based on *t*‐score. (Panel B) Venn diagrams illustrating degree of overlap between genes upregulated/downregulated by chronic alcohol use in the liver and muscle. (Panel C) Bubble plot depicting results from over‐representation analysis of MSigDB mouse Molecular Hallmark and GTRD gene sets for genes commonly/uniquely dysregulated by chronic alcohol use in the liver and muscle (as per Panel B). Circle size is proportional to the number of gene hits as a % of the total number of annotated genes for a given overlap. Red and blue shading denote gene sets significant over‐represented in upregulated and downregulated features, respectively (adjusted *p* ≤ 0.05, enriched for ≥2 genes).

Chronic alcohol markedly perturbed the transcriptomes of both liver and muscle, with over 1000 dysregulated genes in each case (Figure 2A). However, the liver transcriptome was more sensitive to the effects of chronic alcohol, exhibiting more differentially expressed genes and greater magnitudes of change compared with skeletal muscle (Figure 2A). An overlay of dysregulated genes showed that liver and muscle experience largely unique transcriptomic responses to chronic alcohol (Figure 2B). In the liver, uniquely upregulated genes were enriched with MTA3 transcription factor (TF) targets and glycolysis genes, while uniquely downregulated genes were associated with processes, such as cholesterol homeostasis and hypoxia (Figure 2C, Figure S1). In muscle, uniquely upregulated genes largely mapped to inflammatory processes, apoptosis and extracellular matrix processes as well as CRELD1 and LYL1 TF targets, while uniquely downregulated genes were linked to fatty acid metabolism, oxidative phosphorylation and mitochondrial biogenesis, and included targets of the NR1H2 and PPARGC1 TFs (Figure 2C, Figure S1). Fewer genes were commonly dysregulated by chronic alcohol across both tissues, with commonly upregulated genes involved in carbohydrate metabolism processes, and commonly downregulated genes related to amino acid metabolism, chromosome‐related processes, and targets of the AFF4 and RXRG TFs (Figure 2C, Figure S1). Tissue specificity was also evident among the top genes dysregulated by alcohol, with virtually all top genes in liver (except *Arsa*, *Sox12*, and *Paqr7*) unaffected by alcohol in muscle and, similarly, nearly all top genes in muscle (except *Gstk1*, *Ivd*, *Pxmp2*, and *Sertad3*) unaffected by alcohol in the liver (Figure 2A). Top liver‐specific genes included many related to alcohol and/or cholesterol metabolism (*Cyp2d9*, *Cyp3a41b*, *Cyp3a44*, *Cyp51*, *Idi1*, *Msmo1*, *Nsdhl*, *Rdh11*, and *Tkfc*), a glutathione conjugator (*Gstm3*; upregulated) and a leptin receptor (*Lepr*; upregulated). Top muscle‐specific genes included those linked to cytoskeletal regulation (*Actc1*, *Tmsb10*, and *Vasp*; all upregulated), metabolism of pro‐inflammatory mediators (*Cyp4f18*; upregulated) and mitochondrial energetics (*Coq10a* and *Mpc1*; both downregulated) (Figure 2A).

### Dysregulation of the muscle proteome is minimal following chronic alcohol intake

**FIGURE 3:**
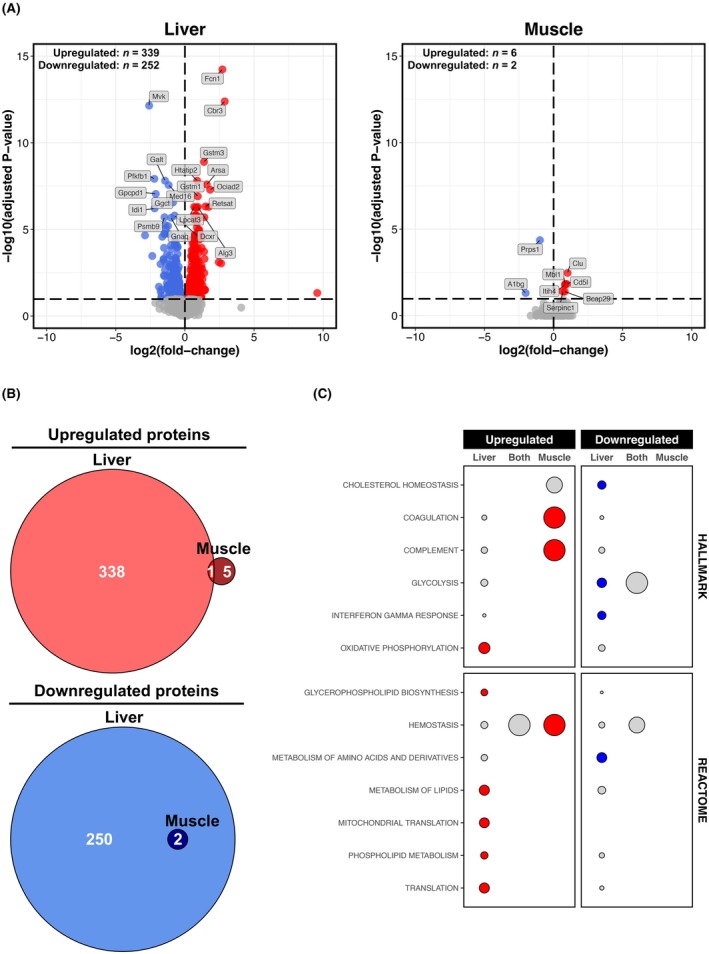
Liver versus muscle proteomic responses to chronic alcohol. (Panel A) Volcano plots for differential protein abundance analysis (Alcohol vs. Control) in the liver and muscle. Red and blue shading denote significant upregulation and downregulation, respectively (adjusted *p* ≤ 0.1). Annotated proteins are those significant proteins ranked in top 10 upregulated/downregulated based on *t*‐score. (Panel B) Venn diagrams showing degree of overlap between proteins upregulated/downregulated by chronic alcohol use in the liver and muscle. (Panel C) Bubble plot depicting results from over‐representation analysis of MSigDB Molecular Hallmark and Reactome Pathways gene sets for proteins commonly/uniquely dysregulated by chronic alcohol use in the liver and muscle (as per Panel B). Circle size is proportional to the number of hits as a % of the total number of annotated genes for a given overlap. Red and blue shading denote gene sets significant over‐represented in upregulated and downregulated features, respectively (adjusted *p* ≤ 0.05, enriched for ≥2 features).

Like the transcriptome, the liver proteome was more sensitive to chronic alcohol than the muscle proteome (Figure 3A). While approximately 600 proteins were dysregulated by alcohol in liver, only eight proteins were dysregulated in muscle (Figure 3A). Overlaying these signatures revealed a major liver‐specific proteome response to chronic alcohol, accompanied by a limited muscle‐specific response and few commonly dysregulated proteins (Figure 3B). In liver, uniquely upregulated proteins were involved in processes, such as oxidative phosphorylation, lipid metabolism, and mitochondrial translation, while uniquely downregulated proteins were associated with cholesterol homeostasis, glycolysis, and amino acid metabolism (Figure 3C). In contrast, proteins uniquely upregulated in muscle were enriched in processes, such as coagulation, hemostasis, and the immune complement system (Figure 3C). No proteins were uniquely downregulated in muscle (Figure 3B), neither were there any enriched terms for commonly dysregulated proteins nor enriched TF target sets in any case (Figure 3C). Unsurprisingly, all top‐dysregulated proteins in the liver were unaffected by alcohol in muscle (Figure 3A), with most (except Alg3, Fcn1, Galt, Gnaq, Med,16, and Psmb9) also being uniquely dysregulated at the gene level in the liver. This included several metabolism‐related proteins (Cbr3, Dcxr, Idi1, Lpcat3, Mvk, Pfkfb1, and Retsat) and two glutathione conjugators (Gstm1 and Gstm3; both upregulated) (Figure 3A). In contrast, top muscle‐specific proteins included stress‐related chaperones (Bcap29 and Clu; both upregulated), blood coagulation regulators (Serpinc1; upregulated), and immune/inflammatory elements (Cd5l and Mbl1; both upregulated) (Figure 3A).

### Transcriptome and proteome responses to chronic alcohol use are more synergistic in the liver compared with muscle

**FIGURE 4:**
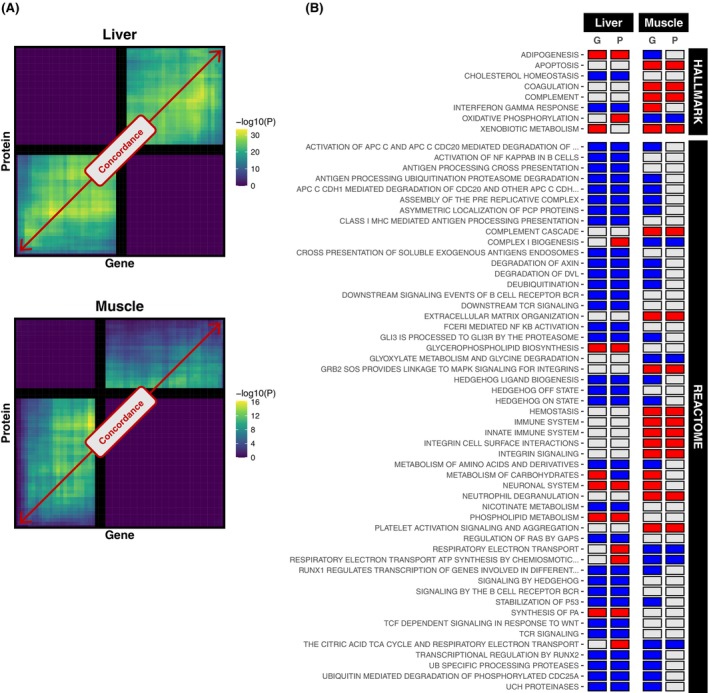
Global comparison between transcriptomic and proteomic responses to chronic alcohol in the liver and muscle. (Panel A) Rank‐rank hypergeometric overlap (RRHO) plots illustrating the degree of correspondence between gene level and protein‐level responses to chronic alcohol use in the liver and in muscle. The lighter the coloring in the lower‐left quadrant, the stronger the concordance in upregulation between genes and proteins. The lighter the coloring in the upper‐right quadrant, the stronger the concordance in downregulation between genes and proteins. For RRHO analysis, only unique features present at both the gene level and protein level were included (*n* = 3303 gene/proteins), with features ranked on *t*‐score. (Panel B) Heatmap comparing global pathway regulation by chronic alcohol use in liver and muscle at gene and protein levels. Shown are those MSigDB mouse Molecular Hallmarks and Reactome Pathways that are similarly regulated at gene and protein levels in at least one of the two tissues. Results were derived via gene set enrichment analysis applied to unique features present at both the gene level and protein level (*n* = 3303 gene/proteins), with features ranked on *t*‐score. Red and blue shading denote significant upregulation (adjusted *p* ≤ 0.1, normalized enrichment score > 0) and downregulation (adjusted *p* ≤ 0.1, normalized enrichment score < 0), respectively. G, gene; P, protein.

With our transcriptomic and proteomic data exhibiting some consistent outcome themes (e.g., quantitative differential patterns and functions of dysregulated features), we further explored the general concordance between gene and protein responses to chronic alcohol in each tissue using “threshold‐free” approaches to maximize global biological signal (Cahill et al., 2018). We observed a strong degree of agreement in the liver between the differential patterns of features present at both the gene and protein levels (*n* = 3303 genes/proteins) (Figure 4A). While a trend for agreement between differential gene and protein patterns was also observed in muscle, the corresponding signal was much weaker compared with that in the liver (Figure 4A). Similarly, the agreement for global pathway regulation between gene and protein levels was more apparent in the liver than in muscle (Figure 4B). These analyses revealed numerous themes in line with our individual transcriptome and proteome analyses. For example, in muscle, there was a strong upregulation of inflammatory (e.g., “neutrophil degranulation,” “innate immune system,” “platelet aggregation signalling and aggregation,” “complement cascade”), extracellular matrix and apoptosis pathways. In the liver, there was a unique downregulation of cholesterol homeostasis pathways. Both liver and muscle showed common downregulation of amino acid metabolism pathways at the gene level, which extended only to the protein level in the liver. Additionally, there were opposing mitochondrial and oxidative phosphorylation responses in the liver compared with the muscle (Figure 4B).

### Metabolome responses to chronic alcohol are less pronounced in muscle than the liver

**FIGURE 5:**
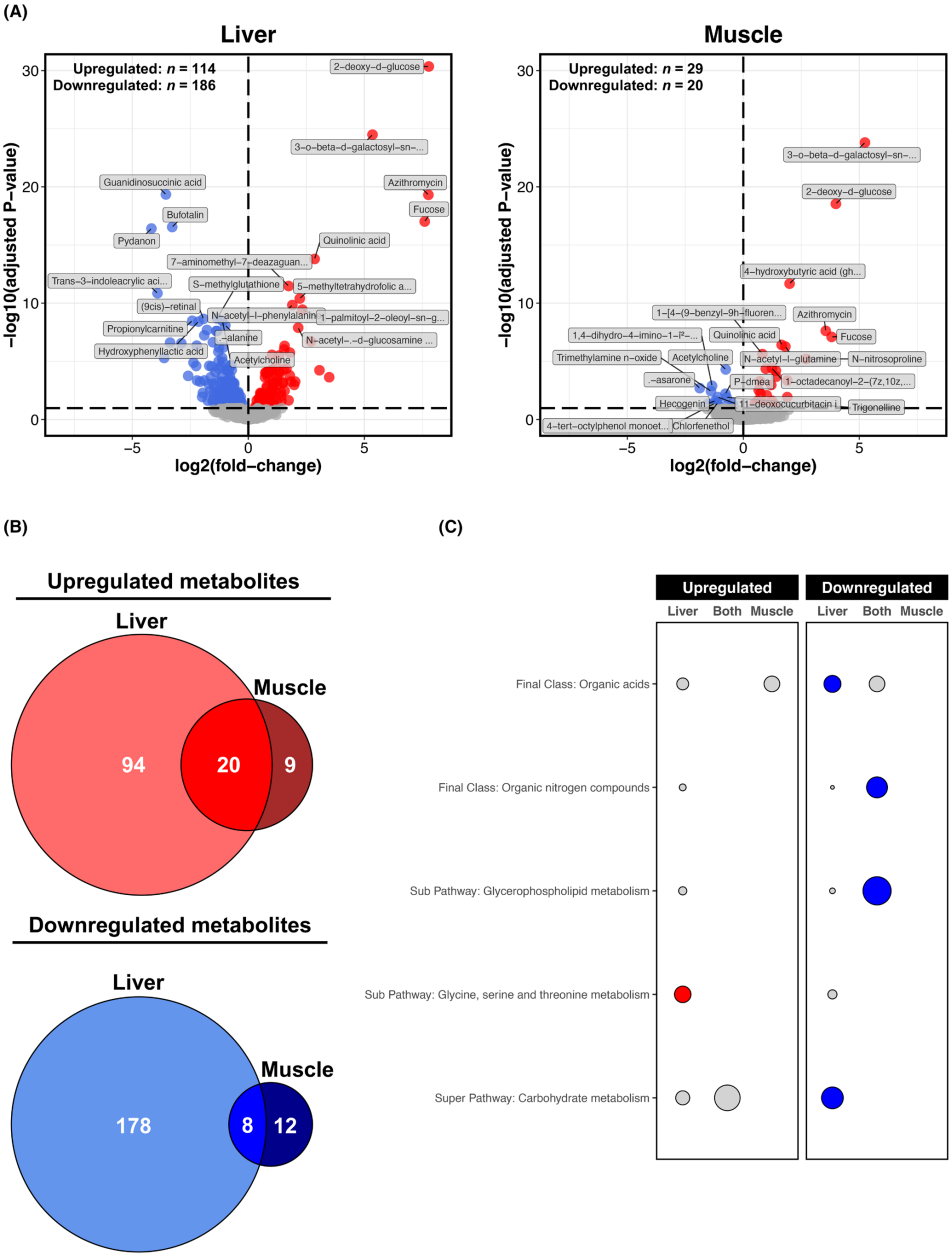
Liver versus muscle metabolomic responses to chronic alcohol. (Panel A) Volcano plots for differential metabolite analysis (Alcohol vs. Control) in the liver and muscle. Red and blue shading denote significant upregulation and downregulation, respectively (adjusted *p* ≤ 0.1). Annotated metabolites are those significant metabolites ranked in top 10 upregulated/downregulated based on *t*‐score. (Panel B) Venn diagrams showing degree of overlap between metabolites upregulated/downregulated by chronic alcohol use in the liver and muscle. (Panel C) Bubble plot depicting results from over‐representation analysis of metabolite final class, super pathway and sub pathway annotations for metabolites commonly/uniquely dysregulated by chronic alcohol use in the liver and muscle (as per Panel B). Circle size is proportional to the number of metabolite hits as a % of the total number of annotated metabolites for a given overlap. Red and blue shading denote significant over‐representation in upregulated and downregulated metabolites, respectively (adjusted *p* ≤ 0.05, enriched for ≥2 metabolites).

As at the transcriptome and proteome levels, the liver metabolome was more sensitive to chronic alcohol than the muscle metabolome, with sixfold more metabolites dysregulated in the liver compared with muscle (Figure 5A). Overlaying differential metabolites revealed a strong liver‐specific response to alcohol, with fewer metabolites either uniquely dysregulated in muscle or commonly dysregulated in both tissues (Figure 5B). In the liver, uniquely upregulated metabolites were strongly associated with the glycine, serine, and threonine metabolism pathway, while uniquely downregulated metabolites were linked to the carbohydrate metabolism pathway and the organic acid class (Figure 5C). Metabolites downregulated by alcohol in both tissues were predominantly organic nitrogen compounds and metabolites involved in glycerophospholipid metabolism (Figure 5C). However, no enriched terms were identified for metabolites either upregulated in both tissues or uniquely dysregulated in muscle (Figure 5C). Interestingly, despite the general tissue specificity of upregulated metabolites (Figure 5B), liver and muscle shared many top‐upregulated metabolites, including 2‐deoxy‐d‐glucose, 3‐o‐beta‐d‐galactosyl‐sn‐glycerol, azithromycin, fructose (all carbohydrate related), and quinolinic acid (pyridine related) (Figure 5A). Acetylcholine was the only top‐downregulated metabolite in both tissues, with all other top‐downregulated metabolites in the liver being metabolites unaffected by alcohol in muscle (Figure 5A). Conversely, only four top‐downregulated metabolites in muscle were not dysregulated in the liver, namely, hecogenin (a terpenoid), cholfenethol (an acaricide), 4‐tert‐octylphenol monoethoxylate (a fatty alcohol), and beta‐asarone (a phenylpropanoid) (Figure 5A). Top liver‐specific metabolites included the upregulated compound (9cis)‐retinal (a retinol‐related compound) and downregulated compounds, such as propionylcarnitine (a fatty acid ester), bufotalin (a steroid), trans‐3‐indoleacrylic acid (an indole), hydroxyphenyllactic acid (a phenylpropanoic acid), beta‐alanine, and guanidinosuccinic acid (both amino acid‐related compounds) (Figure 5A). The identification confidence levels for all analyzed metabolites are detailed in Data S1. These range from Level 1 (highest confidence), assigned to metabolites definitively identified by matching authentic standards, to Level 4 (low confidence), representing unknown compounds or tentative assignments based on database matches without confirmatory spectral or standard‐based validation.

### Liver and muscle lipidomes are similarly sensitive to chronic alcohol consumption

**FIGURE 6:**
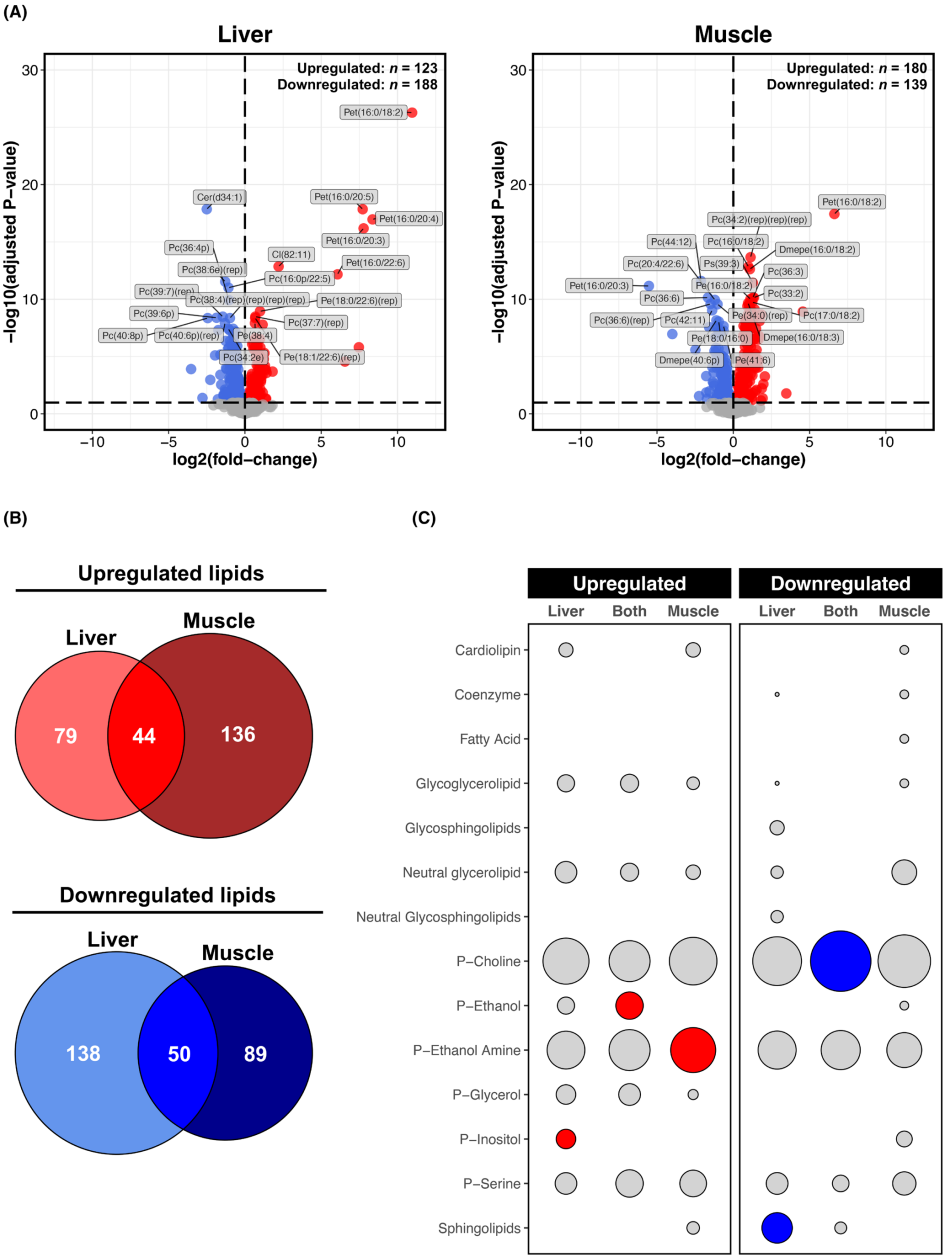
Liver versus muscle lipidomic responses to chronic alcohol. (Panel A) Volcano plots for differential lipid analysis (Alcohol vs. Control) in the liver and muscle. Red and blue shading denote significant upregulation and downregulation, respectively (adjusted *p* ≤ 0.1). Annotated lipids are those significant lipids ranked in top 10 upregulated/downregulated based on *t*‐score. (Panel B) Venn diagrams illustrating degree of overlap between lipids upregulated/downregulated by chronic alcohol use in the liver and muscle. (Panel C) Bubble plot depicting results from over‐representation analysis of main lipid classes for lipids commonly/uniquely dysregulated by chronic alcohol use in the liver and muscle (as per Panel B). Circle size is proportional to the number of lipid hits as a % of the total number of annotated lipids for a given overlap. Red and blue shading denote significant over‐representation in upregulated and downregulated lipids, respectively (adjusted *p* ≤ 0.05, enriched for ≥2 lipids).

Unlike all other omics layers studied, the lipidomes of liver and muscle were comparably sensitive to chronic alcohol, with approximately 320 lipids differentially regulated in each case (Figure 6A). Nevertheless, overlaying lipidome profiles revealed large tissue‐specific lipid responses to alcohol, although noticeable proportions of commonly dysregulated lipids were also found (Figure 6B). Commonly upregulated lipids were enriched with P‐ethanol compounds, while commonly downregulated lipids were enriched with P‐choline compounds (Figure 6C). Lipids uniquely upregulated in the liver predominantly belonged to the P‐inositol class, whereas those uniquely downregulated in the liver were enriched with sphingolipids (Figure 6C). Lipids uniquely upregulated in muscle strongly mapped to the P‐ethanol Amine class, although no enriched classes were identified for lipids uniquely downregulated in muscle (Figure 6C). Among the top‐downregulated lipids in muscle were P‐choline, P‐ethanol, and P‐ethanol Amine compounds that were either upregulated (Pc(36:6)(rep), Pet(16:0/20:3)), or unperturbed (Pc(36:6), Pc(44:12), Dmepe(40:6p)) by alcohol in liver (Figure 6A). Similarly, half of the top‐upregulated lipids in muscle were compounds downregulated by alcohol in the liver (Pc(16:0/18:2), Pc(17:0/18:2), Pc(33:2), Pc(36:3), Ps(39:3)) (Figure 6A). In both liver and muscle, the top‐upregulated lipid was Pet(16:0/18:2), a P‐ethanol compound and biomarker of recent alcohol consumption (Aboutara et al., 2023) (Figure 6A).

### Multi‐omic networks discriminate chronic alcohol use in the liver and in muscle

**FIGURE 7:**
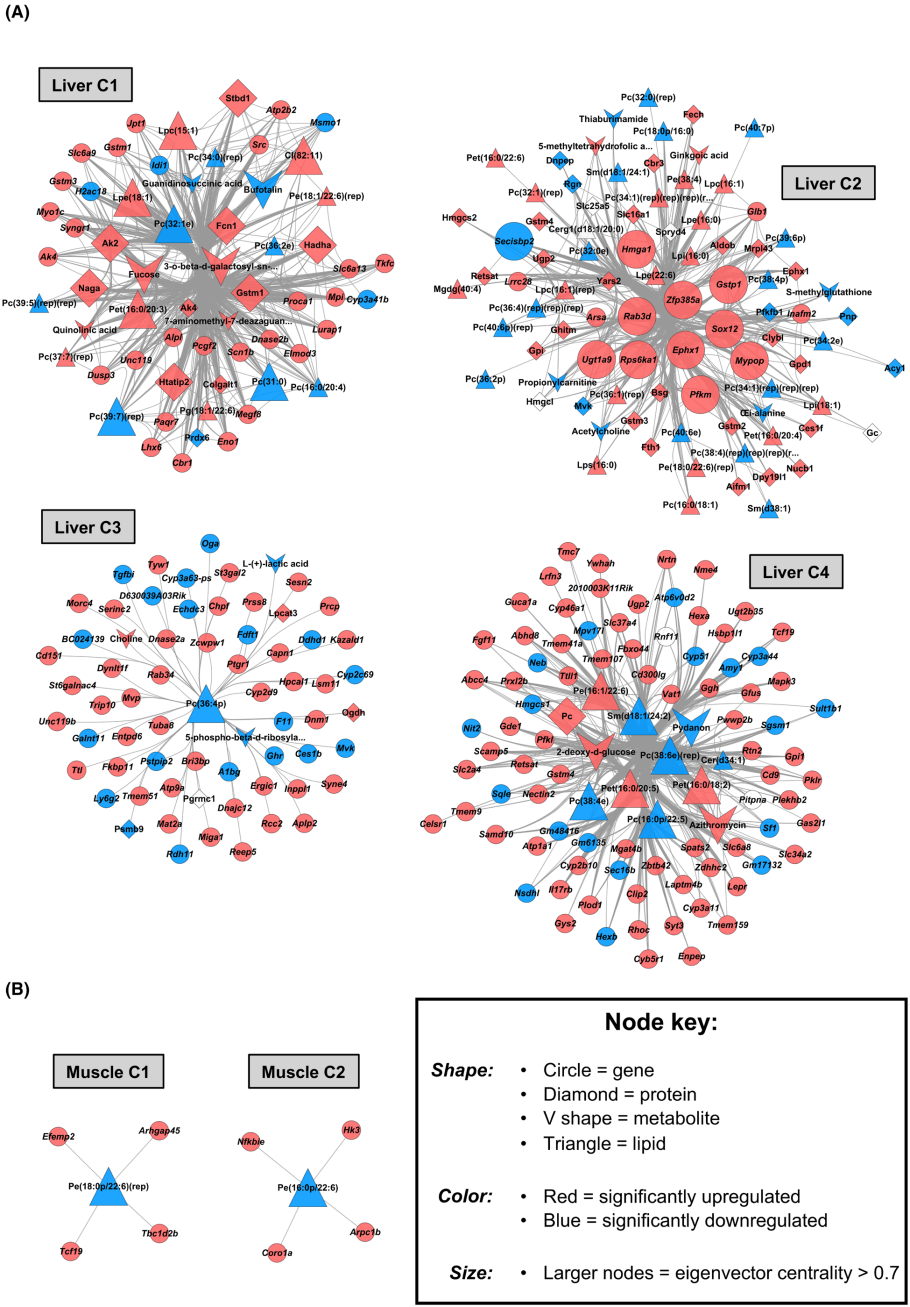
Integrative multi‐omic network modeling of the liver and muscle responses to chronic alcohol. Communities of strongly connected molecular features in the liver (Panel A) and muscle (Panel B) with chronic alcohol use, as derived via Mixomics DIABLO analyses with downstream correlation network generation (|correlation coefficient| > 0.85, with component 1 used for the liver and component 2 used for muscle).

**FIGURE 8:**
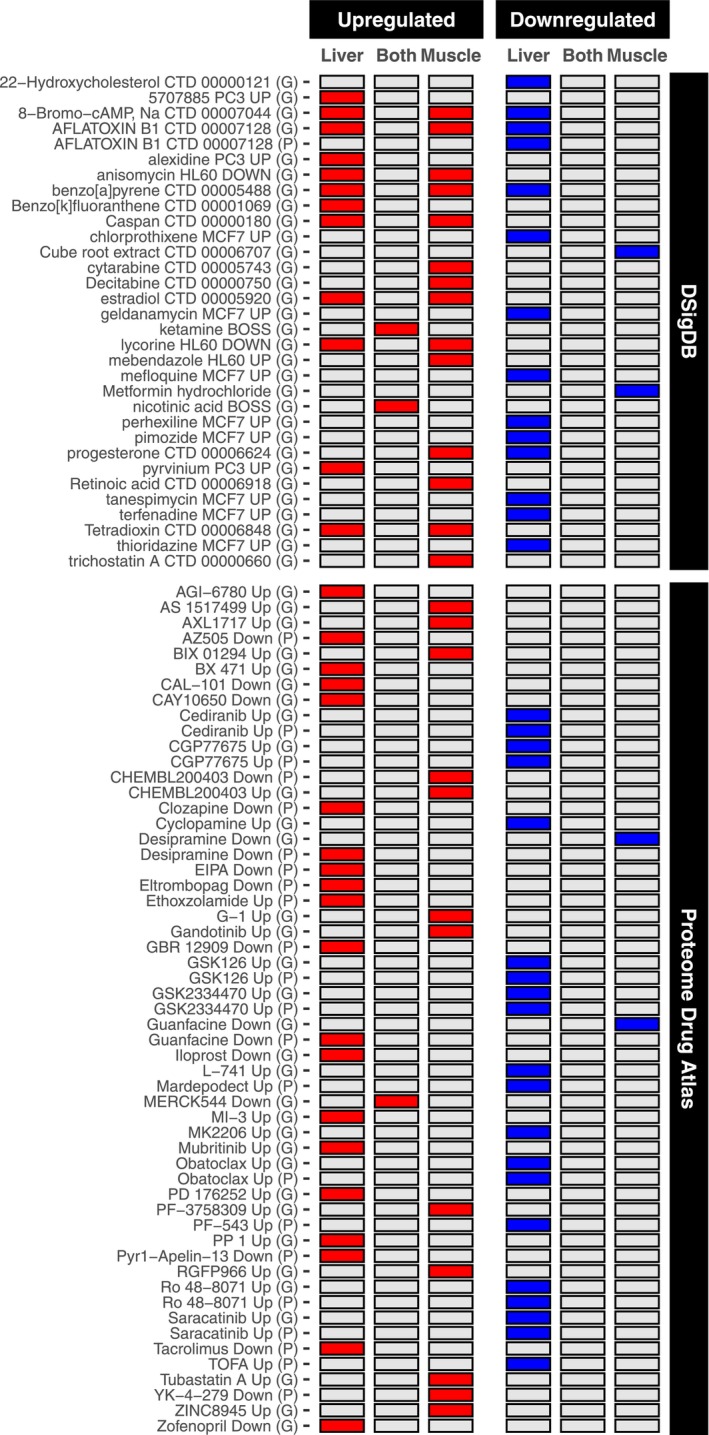
Omics‐driven repurposing of drug therapeutics for alcohol‐related liver disease (ALD) and alcohol‐related myopathy. Heatmap includes results from over‐representation analysis of human Drug Signature Database (DSigDB) and Proteome Drug Atlas sets for genes/proteins commonly/uniquely dysregulated by chronic alcohol use in the liver and muscle. Shown are the top 10 most significant sets for each common/unique feature permutation. Red and blue shading denote significant over‐representation in upregulated and downregulated genes/proteins, respectively (adjusted *p* ≤ 0.05, enriched for ≥2 features). G, enriched when genes used as input; P, enriched when proteins used as input.

We also performed discriminative multi‐omic network analysis to model complex relationships across the different omics layers of molecular biology and, in turn, establish further key molecular drivers of alcohol‐induced muscle/liver pathophysiology. In the liver, a multi‐omic relevance network of 309 features was deduced, comprising a mix of genes (*n* = 187), proteins (*n* = 48), metabolites (*n* = 19), and lipids (*n* = 55). The liver relevance network partitioned into four subnetworks of varying size (Liver C1–C4), each containing at least one feature from each omics type (Figure 7A). Virtually all (*n* = 301) features of the liver relevance network showed dysregulation by chronic alcohol in the liver (Figure 7A). Hub analyses further defined a list of 40 “priority” features across the liver network, including 11 genes, 8 proteins, 6 metabolites, 15 lipids (Figure 7A). Many of these upregulated features were related to metabolism and energy homeostasis, including: the genes *Ephx1* (lipid metabolism), *Pfkm* (glycolysis), and *Zfp385*a (adipogenesis) (Liver C2 hubs); the proteins Ak2 (energy homeostasis), Hadha (mitochondrial beta‐oxidation), Htatip2 (redox sensor), Stbd1 (cargo receptor for glycogen) (Liver C1 hubs), and Pc (glucose and lipid synthesis) (Liver C4 hub), and; the metabolites 3‐o‐beta‐d‐galactosyl‐sn‐glycerol (carbohydrate metabolism), fucose (carbohydrate class) (Liver C1 hubs), 2‐deoxy‐d‐glucose (carbohydrate class), and azithromycin (carbohydrate class) (Liver C4 hubs) (Figure 7A). Several upregulated features related to conjugation were also identified among liver hubs, including *Ugt1a9* (glucuronidation pathway) (Liver C2 hub) and the reduced glutathione conjugators *Gstp1* (Liver C2 hub) and Gstm1 (Liver C1 hub) (Figure 8A). Additionally, more than half of liver hub lipids were downregulated P‐choline compounds, including Lpc(15:1), Pc(31:0), Pc(32:1e), Pc(39:7)(rep) (Liver C1 hubs), Pc(16:0p/22:5), Pc(38:4e), Pc(38:6e)(rep) (Liver C4 hubs), and the sole hub of Liver C3, Pc(36:4p) (Figure 7A). Pet(16:0/18:2) (P‐ethanol compound), the top‐upregulated lipid in liver (Figure 7A), was also found among the hubs of Liver C4 (Figure 7A).

Compared with the liver, the muscle multi‐omic relevance network was much smaller and contained only genes (*n* = 8; all downregulated by alcohol in muscle) and lipids (*n* = 2; both upregulated by alcohol in muscle). The muscle relevance network partitioned into two subnetworks (Muscle C1 and C2), each comprising one lipid and four genes (Figure 7B). Muscle C1 contained upregulated genes related to transcription (*Tcf19*), endocytosis (*Tbc1d2b*), collagen fibril assembly (*Efemp2*), and actin cytoskeleton regulation (*Arhgap45*), all centered around Pe(18:0p/22:6)(rep), a downregulated P‐ethanol Amide compound and the sole hub feature within Muscle C1 (Figure 7B). Muscle C2 followed a similar topology, with upregulated genes related to NF‐Kappa‐B inhibition (*Nfkbie*), hexose phosphorylation (*Hk3*), and actin cytoskeleton dynamics (*Arpc1b* and *Coro1a*) all converging on one downregulated “hub” P‐ethanol Amide compound, Pe(16:0p/22:6) (Figure 7B).

### Chronic alcohol use associates with omic profiles that are targetable by drugs

Given the need to establish new therapeutics against ALD and alcohol‐related myopathy, we screened for potential drug treatments in humans based on the omic signatures derived in this study. We specifically focused on transcriptomic and proteomic profiles due to the greater availability of drug‐target annotations compared with the metabolome and lipidome. Consequently, a total of 745 compounds from the Drug Signature Database (Yoo et al., 2015) and Proteome Drug Atlas (Mitchell et al., 2023) were predicted to target features dysregulated by chronic alcohol in liver and/or muscle. Consistent with the general tissue specificity observed in transcriptome and proteome responses to alcohol, virtually all (*n* = 742) compounds were predicted to target features uniquely dysregulated by chronic alcohol in either liver or muscle. This trend remained evident even when considering the top drug compound predictions based on significance (Figure 8). Among the predicted compounds for liver‐specific features, several were reproducibly identified across transcriptome and proteome inputs. These included Saracatinib and GSK126, which may potentially hold antiliver fibrotic properties (Seo et al., 2020; Zhang et al., 2023) (Figure 8). Predicted compounds for features uniquely dysregulated by alcohol in muscle included metformin, trichostatin A, and retinoic acid, each of which may positively impact the phenotype observed with alcohol‐related myopathy (Dupre‐Aucouturier et al., 2015; Lamarche et al., 2015; Shang & Miao, 2023) (Figure 8). Only three compounds were predicted for features commonly upregulated by chronic alcohol in both liver and muscle, namely, ketamine, nicotinic acid, and MERCK544, while no compounds were predicted for features commonly downregulated by chronic alcohol in both tissues (Figure 8). However, several compounds, such as estradiol and lycorine, were predicted to target unique responses in both tissues (Figure 8), indicating that some compounds may impact both liver and muscle after alcohol intake, but through distinct mechanisms. The presence of predicted compounds with known carcinogenic potential (e.g., aflatoxin B1, benzo[a]pyrene) (Figure 8) may suggest that chronic alcohol exposure contributes to metabolic alterations with possible toxicological implications for both liver and skeletal muscle. The drug targets altered by alcohol are listed in Data S1.

## DISCUSSION

Among the most serious and prevalent consequences of excessive alcohol consumption are ALD and alcohol‐related myopathy. However, therapeutic options are currently lacking, underscoring the urgency for continued research into the molecular blueprints of alcohol‐induced liver and muscle pathophysiology. Multi‐omics has emerged as a cutting‐edge frontier for accelerating molecular understanding of and developing countermeasures against complex diseases. Therefore, we undertook the first multi‐omic screen of chronic alcohol signatures in liver versus skeletal muscle. Our findings revealed that liver and muscle are characterized by unique molecular profiles in the context of chronic, excessive alcohol intake. Consequently, liver and muscle from mice that consumed alcohol were associated with mostly divergent therapeutic targets and candidate pharmacologic interventions. These results have important clinical implications for developing optimal strategies to ameliorate ALD and alcohol‐related myopathy both individually and synergistically.

Mechanistic investigations of chronic alcohol drinking in people come with significant ethical and technical challenges, highlighting the importance of preclinical studies that mimic human responses to excessive alcohol consumption. In the current study, mice that drank 20% alcohol daily for 34–40 weeks had less muscle mass and were weaker than control mice, indicative of alcohol‐induced myopathy (Moser et al., 2022, 2023). Gold‐standard clinical markers of ALD include elevated serum levels of AST and ALT (Hall & Cash, 2012), which reflect their leakage from hepatocytes upon alcohol‐induced injury. While serum AST and ALT levels were not quantified, it was reported that people with lower hepatic levels of GOT1 (AST) and GPT (ALT) experienced more severe ALD (Niu et al., 2022), suggesting that declines in AST and ALT levels in liver tissue itself may reflect development of ALD. Consistent with this, mice consuming alcohol had diminished levels of liver Got1 and Gpt (Data S1), as well as a lower hepatic PC:PE ratio, which is a hallmark of impaired hepatocyte membrane integrity that leads to progression of steatosis into steatohepatitis (Li et al., 2006) and is indicative of ALD in both patients and animal models (Jeon & Carr, 2020). Thus, while we acknowledge that this omics‐based study would have benefited from liver histology to confirm the presence, progression and/or severity of ALD, the observations recently noted offer some support to the validity of our model for studying alcohol‐induced dysregulation of both liver and skeletal muscle at the molecular level.

The liver metabolizes over 90% of absorbed alcohol, making it highly susceptible to the toxic effects of chronic alcohol intake (Hyun et al., 2021; Osna et al., 2017). Greater molecular disruption by chronic alcohol in the liver compared with muscle is therefore logical. Consistent with this notion, we found that the liver is more sensitive than muscle to chronic alcohol at the levels of the transcriptome, proteome, and metabolome. Widespread changes across the liver transcriptome, proteome, and metabolome have also been observed in human ALD models (Massey et al., 2021; Niu et al., 2022), although omics work in myopathic human muscle is lacking. Conversely, the lipidomes of liver and muscle were found to be equally susceptible to chronic alcohol consumption. It is well established that alcohol causes gross dysregulation of liver lipids (Jeon & Carr, 2020). Moreover, previous human lipidomics research noted that wholesale hepatic lipidome changes associate with alcohol‐related liver injury (Thiele et al., 2023). We therefore postulate that liver and muscle lipidomes exhibit similar sensitivity to chronic alcohol due to a relative increase in muscle lipidome sensitivity rather than a decline in liver lipidome sensitivity. This could be due to several factors. First, it is plausible that remodeling of the muscle lipidome after chronic alcohol intake resulted from lipid “spillover” from other sources (e.g., adipose tissue) that were then transported into muscle. Second, it is possible that alcohol directly modified muscle lipids. Indeed, many upregulated lipids in muscle (and liver) were phosphatidylethanols, which only form in the presence of ethanol (Aboutara et al., 2023). Lastly, the lipidome provides a snapshot of processes across the genome, transcriptome, and proteome, so cumulative changes across those layers could predispose muscle to gross lipidome remodeling. Taken together, such data indicate that lipid composition in skeletal muscle is highly influenced by chronic alcohol consumption.

Another interesting observation was that there were few individual protein changes in muscle after chronic alcohol intake, even though a substantial number of genes were found to be dysregulated. This lack of individual protein changes relative to gene changes may be due to a “bulk” impairment in translational efficiency, similar to what occurs in other long‐term models of muscle decline, such as aging (Roberts et al., 2010). Another plausible explanation is that muscle exhibits a “biphasic” adaptive temporal response to chronic alcohol intake, such that the molecular signatures captured at our end time point reflect the onset of “later” molecular mechanisms of alcohol‐related myopathy which are initially prominent at the transcriptome level. Alternatively, it may be that mRNA processing undergoes “fine‐tuning” in myopathic muscle once protein levels converge on a “set point” as rates of muscle decline subdue over time (Ganjayi et al., 2023). In this scenario, larger changes in key mRNA could gradually couple to subtle protein‐level changes that still contribute to alcohol‐related myopathy. Supporting this notion, we observed a strong concordance between the transcriptome and proteome in *whole‐pathway* changes related to muscle maintenance/function after chronic alcohol intake. Thus, heavy alcohol drinking is characterized by a more “global” and persistent “steady‐state” mRNA‐protein response in the liver than in skeletal muscle. This observation may reflect temporal differences in the onset and progression of ALD versus alcohol‐related myopathy which might, in turn, have implications for developing optimal therapeutics for each tissue. We also acknowledge that the abundance of myofibrillar proteins can limit the sensitivity to detect differences in less abundant skeletal muscle proteins.

Arguably the most pertinent finding in the current study was that the molecular profiles induced in the liver and muscle by chronic alcohol are mostly unique to one another, implying that largely distinct mechanisms characterize ALD and alcohol‐related myopathy. The molecular profile of the liver was mainly defined by an extensive and multi‐layered metabolic remodeling signature, encompassing changes related to lipid, carbohydrate, and glucose/glycogen metabolism that were not observed in muscle. Extensive metabolic reprogramming has also been witnessed in human ALD liver tissue omics (Massey et al., 2021), and our findings suggest that liver and muscle experience distinct metabolic reprogramming events due to chronic alcohol drinking. Widespread metabolic dysregulation is a hallmark feature of alcohol‐induced liver damage. Indeed, the majority of absorbed alcohol is oxidized in the liver to acetate, during which aberrant changes in hepatic metabolism across glycolytic, gluconeogenic, and fatty acid pathways occur, exacerbating the ALD phenotype (Cunningham & Van Horn, 2003; Wilson & Matschinsky, 2020). Congruent with liver transcriptomics in ALD patients (Wruck & Adjaye, 2017), cholesterol homeostasis was strongly downregulated in the present study. Moreover, we found that members of the *Cyp3a* gene family, which play key roles in metabolism, were among the most significantly downregulated by alcohol in the liver. This aligns with observations from human omics studies of ALD, which report decreased hepatic CYP3A4 levels (Prasad et al., 2018; Wruck & Adjaye, 2017).

Molecular profiles related to mitochondrial translation and oxidative phosphorylation were noted to be uniquely upregulated by alcohol in the liver, supporting the notion that enhanced mitochondrial respiration in the liver due to alcohol consumption is associated with greater injury and damage (Han et al., 2012). Transcriptomics data in ALD patients also point to elevated oxidative phosphorylation gene expression in peripheral blood (Yang et al., 2022). Elevated hepatic mitochondrial respiration with chronic alcohol may reflect an adaptive response to enhance liver alcohol metabolism or could signify chronically “overworked” mitochondria. The latter would lead to an increased mitochondrial reactive oxygen species (ROS) production (Hoek et al., 2002), which is implicated as a key mechanism in ALD (Garcia‐Ruiz et al., 2013). Consistent with this, Glutathione S‐transferase (GST) enzymes (Gstm1, Gstm3, and Gstp1) were among the top uniquely upregulated molecules in the liver of mice that consumed alcohol. Elevated hepatic GSTP1 abundance has recently been uncovered as a strong candidate biomarker of ALD progression (Niu et al., 2022) with meta‐analyses also demonstrating that people with *GSTM1* and *GSTP1* allelic variations hold increased susceptibility to ALD (Marcos et al., 2011). Interestingly, we also noticed Fcn1 among the top‐upregulated proteins by chronic alcohol specifically in liver, consistent with recent hepatic proteomic observations in patients with severe ALD that may be linked to inflammation (Taiwo et al., 2024). Extending previous studies on liver damage (Li et al., 2019; Wang et al., 2017), our data also revealed MTA3 as a promising new metabolism‐related therapeutic target of ALD for future mechanistic investigation.

In contrast to the liver, skeletal muscle exhibited a molecular profile indicative of impaired mitochondrial energetics, consistent with recent integrated multi‐omics analyses reported by Welch and colleagues (Welch et al., 2025). Moreover, it displayed a distinct pattern characterized primarily by elevated inflammation and extensive cytoskeletal and ECM remodeling. Chronic inflammation and mitochondrial dysfunction can both be deemed central tenets in the etiology of muscle atrophy and weakness, potentially contributing to alcohol‐related myopathy (Bonaldo & Sandri, 2013; Hyatt & Powers, 2021; Welch et al., 2025). Cytoskeletal/ECM alterations are also a major component of skeletal muscle remodeling (Goody et al., 2015). An upregulated matrisome profile in muscle after chronic alcohol intake has been suggested to reflect a profibrotic phenotype (Simon et al., 2017). Conversely, many matrisome features upregulated by alcohol in the present work may, in fact, be upregulated to maintain or improve muscle mass and function (Deane et al., 2021). Thus, whether these muscle matrisome changes truly represent a profibrotic phenotype, or instead reflect a compensatory mechanism to avoid advanced muscle wasting with prolonged alcohol use, warrants further determination. Nonetheless, our analysis pinpoints LYL1 (an inflammatory regulator), PPARGC1a (a regulator of mitochondrial biogenesis), and CRELD1 (a matrisome member) TFs as key mechanistic targets of alcohol‐related myopathy.

It is intriguing to consider how the aforementioned findings parallel the extensive remodeling of the skeletal muscle lipidome following chronic alcohol intake. On the one hand, inflammation and mitochondrial dysregulation are prime events that can influence muscle lipid dynamics (Glass & Olefsky, 2012; Montgomery & Turner, 2015). Yet, the lipidome alterations we found appear underscored by wholesale changes in phospholipids, particularly PC and PE species. Muscle phospholipids have been linked to declines in muscle size and strength (Selathurai et al., 2019), albeit via unknown mechanisms, and PC:PE dynamics are implicated in promoting mitochondrial dysfunction and inflammation (Grapentine & Bakovic, 2019; Lee et al., 2018). Phospholipids are also integral components of the sarcolemma, suggesting that muscle PC:PE ratio may be inherently linked to cytoskeletal/ECM remodeling. Thus, our current findings support our recent postulation that remodeling of muscle phospholipid composition may play a role in the etiology of alcohol‐related myopathy (Ganjayi et al., 2024). Indeed, network modeling uncovered two multi‐omic subnetworks in muscle after alcohol intake, both of which had a central “hub” phospholipid connected to cytoskeletal/ECM, inflammation and/or mitochondria‐related transcripts. Coined the “lipotranscriptome,” lipid regulation of the transcriptome is emerging as a promising target for the discovery and development of disease diagnostics and therapeutics (Wang et al., 2022). Alcohol‐induced changes in muscle lipotranscriptome that center on phospholipid regulation of mitochondria, inflammation and/or the matrisome may therefore be among the strongest candidates for understanding and treating chronic alcohol‐related myopathy.

Traditional drug discovery relying on lab‐based compound screens is cumbersome, costly, labor‐intensive, and high‐risk. Omics circumvents many of these barriers to accelerate the drug discovery process by enabling in silico drug repurposing on an unprecedented scale. Exploiting this approach, we used human drug signature databases to computationally predict a refined list of compounds that target molecular profiles of chronic alcohol‐related liver and/or muscle. While the interpretation of all predicted compounds is beyond the scope of this discussion, this list provides a useful tool to expedite future hypothesis‐driven work on ALD and alcohol‐related myopathy therapeutics. For example, among the most prevalent compounds predicted to target the unique molecular profile of chronic ALD were saracatinib and GSK126. Saracatinib has been reported to attenuate liver fibrosis by preventing activation of hepatic satellite cell activation (Du et al., 2020; Seo et al., 2020), while GSK126 also appears to have antifibrotic liver properties (Zhang et al., 2023) and decreases liver fatty acid content (Wu et al., 2018). These two compounds may thus offer promising avenues for exploration in the context of treating ALD. We also identified metformin and trichostatin A as strong candidate therapeutics for alcohol‐related myopathy. Indeed, trichostatin A has been shown to ameliorate muscle atrophy induced by unloading (Dupre‐Aucouturier et al., 2015) and to reduce muscle fatty acid infiltration (Liu et al., 2021). While the muscle therapeutic potential of metformin is somewhat equivocal, evidence indicates that it could help counter muscle decline in pathological scenarios, as would be excessive chronic alcohol drinking, potentially by normalizing mitochondrial dysfunction or disturbed energy metabolism (Shang & Miao, 2023). Furthermore, compounds that target the molecular profiles of both alcohol‐related muscle and liver might represent the most viable concurrent treatments for ALD and alcohol‐related myopathy. Notably, MERCK544 may be a viable compound for dual‐therapeutic targeting of ALD and alcohol‐related myopathy by mitigating 11β‐HSD1‐dependent metabolic dysregulation related to both liver and muscle (Chapman et al., 2013; Loerz & Maser, 2017).

In summary, we undertook the first multi‐omic screening of liver versus skeletal muscle responses to chronic alcohol use. Our results provide several new insights into therapeutic targets and potential pharmacologic interventions for ALD and alcohol‐related myopathy. That said, multi‐omics datasets require cautious interpretation: transcriptomic and proteomic signals can diverge, so mRNA changes should not be assumed to translate directly into protein abundance or activity. Furthermore, our workflows did not evaluate posttranslational modifications (e.g., phosphorylation, acetylation, and glycosylation), which are critical regulators of protein function, localization, and stability; this limits the mechanistic resolution of our proteomic findings. Consequently, comprehensive follow‐up investigations remain crucial to validate these mechanistic lines of inquiry and to assess the true efficacy of our predicted drug compounds in mitigating alcohol‐induced liver and muscle maladaptations. Such work should include tracking the time course of multi‐omic responses to chronic alcohol in both sexes and incorporate a wider range of physiological and morphological readouts (e.g., liver histological analysis to assess stage of liver injury) to provide a more complete picture of the mechanisms underpinning the onset and progression of ALD and alcohol‐related myopathy. Thorough examination of predicted drug compounds, including optimal dosing, delivery strategies, and safety, is also warranted at both the individual tissue and cross‐tissue levels. Overall, our current findings provide a strong benchmark for expediting the mechanistic understanding of ALD and alcohol‐related myopathy in humans and may help accelerate the development of optimal personalized countermeasures.

## AUTHOR CONTRIBUTIONS

Conceptualization: C.R.G.W. and C.W.B. Mouse experimental procedures: M.S.G., A.M.B., S.E.M., and C.W.B. Data analysis; figures and visualization: C.R.G.W. Manuscript original draft: C.R.G.W. and C.W.B. Manuscript review, editing and approval: C.R.G.W., M.S.G., A.M.B., S.E.M., N.J.S., B.C.C., and C.W.B.

## FUNDING INFORMATION

C.R.G.W. and C.W.B. acknowledge support from the UK Research and Innovation Doctoral Career Development Fund scheme, which helped to fund this work. This work was also funded, in part, through the University of Exeter's Project ADA (Accelerating Data Science and Artificial Intelligence). N.J.S. acknowledges the support of the Osteopathic Heritage Foundation through funding for the Osteopathic Heritage Foundation Ralph S. Licklider, D.O., Research Endowment in the Heritage College of Osteopathic Medicine. B.C.C. acknowledges the support of the Osteopathic Heritage Foundation through funding for the Osteopathic Heritage Foundation Harold E. Clybourne, D.O., Endowed Research Chair in the Heritage College of Osteopathic Medicine. C.W.B. acknowledges the support of the Osteopathic Heritage Foundation through funding for the Osteopathic Heritage Foundation Ralph S. Licklider, D.O., Endowed Faculty Fellowship in the Heritage College of Osteopathic Medicine. This work was also supported, in part, by grants from the National Institutes of Health (R01AG044424 and R01AG067758 to B.C.C.).

## CONFLICT OF INTEREST STATEMENT

B.C.C. reports that he has received grants in the past 5 years related to muscle health from the National Institutes of Health, Astellas Global Development Inc., RTI Solutions, Myolex, and NMD Pharma, that he has received personal fees from Regeneron Pharmaceuticals and the Gerson Lehrman Group, that he has received grants from OsteoDx Inc. not related to the submitted work, and that he also serves as co‐founder and scientific director for OsteoDx Inc. The other authors declare that they have no conflicts of interest.

## ETHICS STATEMENT

Experimental procedures were approved by the Ohio University Animal Care and Use Committee (no.: 20‐H‐015). All methods were performed in accordance with the relevant guidelines and regulations. The study is reported in accordance with ARRIVE guidelines.

## Supporting information


Appendix S1.

Data S1.

Figure S1.

## Data Availability

Raw transcriptomic data from current study are deposited in the NCBI Sequence Read Archive repository (https://www.ncbi.nlm.nih.gov/sra) with links to BioProject ID PRJNA1274880. Raw proteomic data from current study is deposited in the EBI Proteomics Identification Database repository (https://www.ebi.ac.uk/pride/) under the dataset identifier PXD064997. Raw metabolomic and lipidomic data are deposited in the EBI MetaboLights repository (https://www.ebi.ac.uk/metabolights/) under the study identifier MTBLS12616. The data reported in the manuscript itself are provided in Data S1.
