# Supplementary material for: Comparative multi‐omics in female mice reveals tissue‐specific vulnerabilities to chronic alcohol intake
Source: Alcohol Clin Exp Res (Hoboken). 2026 Jan 30;50(2):e70240. doi: 10.1111/acer.70240 (PMC12856532; doi:10.1111/acer.70240)
Supplement: Supplementary file 1 — Appendix S1. [file ACER-50-0-s001.docx]

**Supplementary document 1; Omics based statistical analyses**

*Omics differential expression analysis:* Differential analysis was undertaken at the level of each individual omic strand using the limma R package (v3.56.2; <https://doi.org/doi:10.18129/B9.bioc.limma>)(Ritchie et al., 2015). Briefly, linear mixed effects models were fitted with condition (Alcohol, Control) as a ‘fixed’ effect and mouse ID as a ‘random’ effect, utilising the developers’ recommended *duplicateCorrelation* approach. The empirical Bayes method was then used to calculate moderated *t*-scores(Smyth, 2004), and comparisons between Alcohol and Control mice per tissue extracted. For each omics layer, features with a Benjamini-Hochberg adjusted *P*-value ≤ 0.1 were defined as being differentially regulated by chronic alcohol. Differentially regulated features were then overlaid to identify features commonly or uniquely dysregulated by chronic alcohol in liver *versus* muscle.

*Omics over-representation analysis of pathways, TF targets and molecular classes:* Features commonly or uniquely regulated by chronic alcohol in the liver *versus* muscle were subject to over-representation analyses of pathways, TF targets and molecular classes using the clusterProfiler R package (v4.8.3; <https://doi.org/doi:10.18129/B9.bioc.clusterProfiler>) *enrichr* function(Wu et al., 2021). For transcripts and proteins, analyses were performed against MSigDB (v2023.1)(Castanza et al., 2023) mouse Molecular Hallmark, Reactome Pathway and TF target (GTRD) gene sets. Default values for minGSSize and maxGSSize arguments were used, except for GTRD sets, where maxGSSize was set unbounded. For metabolites, analyses were performed against final class, super pathway and sub pathway sets as assigned during metabolite identification (minGSSize = 3, maxGSSize unbounded). For lipids, analysis was performed against lipid main class sets as assigned during lipid identification (minGSSize = 3, maxGSSize unbounded). For each omic strand, the corresponding background list contained all annotated features utilised during differential testing. Over-represented sets were selected as those with a Benjamini-Hochberg corrected *P*-value ≤ 0.05 that were enriched for at least 2 given features.

*Rank-based analysis across the transcriptome and proteome:* The rank-rank hypergeometric overlap method(Cahill et al., 2018) was employed (via the RRHO2 R package, v1.0; <https://github.com/RRHO2/RRHO2>) to decipher the general degree of correspondence between differential gene expression and protein abundance patterns in the liver and in muscle. The algorithm was applied to unique features present at both the gene level and protein level (*n* = 3,303 genes/proteins), with features ranked by *t*-score. Gene set enrichment analysis was also employed as a rank-based method to elucidate global pathway regulation at the gene level and protein level in the liver and in muscle. These analyses were performed using the clusterProfiler R package (v4.8.3; <https://doi.org/doi:10.18129/B9.bioc.clusterProfiler>) *GSEA* function against MSigSB Molecular Hallmark and Reactome Pathway gene sets as above (default minGSSize and maxGSSize argument values). In each case, the algorithm was applied to unique features present at both the gene level and protein level (*n* = 3,303 genes/proteins), with features ranked by *t*-score and enrichment defined at the Benjamini-Hochberg corrected *P*-value ≤ 0.1 level.

*Multi-omic relevance network analysis:* Features from all omics layers were integrated using multi-omic correlation network analysis with the Mixomics R package (v6.24.0; <https://doi.org/doi:10.18129/B9.bioc.mixOmics>) DIABLO method(Singh et al., 2019). Multiblock integration with projection to latent structure models with discriminant analysis was performed, treating each omic strand as a block and condition as the discriminator. A design matrix was used to maximise the strength of relationships between blocks. Relevance networks were extracted for strongly correlated features across omics layers (|correlation coefficient| > 0.85). Component 1 was chosen for association estimates in the liver and component 2 for muscle, based on inspection of individual sample plots and loading weights. Sub-networks of each relevance network were determined via multi-level community analysis using the igraph R package (v1.5.1; <https://r.igraph.org/>)(Csardi, 2006), with |correlation coefficient| used as edge weight. Highly connected ‘hub’ features of each sub-network were defined with an eigenvector centrality score > 0.7. Networks were visualised using Cytoscape (v3.10.0; <https://cytoscape.org/>)(Shannon et al., 2003).

*Omics-driven drug prediction analysis:* Transcriptome and proteome features commonly or uniquely dysregulated by chronic alcohol in the liver *versus* muscle were further subjected to over-representation analyses against human Drug Signature Database(Yoo et al., 2015) and Proteome Drug Atlas(Mitchell et al., 2023) sets using the Enrichr online webtool (<https://maayanlab.cloud/Enrichr/>)(Chen et al., 2013). For each omic strand, the background list contained all annotated features utilised during differential testing. Ortholog conversions were handled implicitly as part of the built-in functionality of the Enrichr tool. Over-represented sets were selected as those with a Benjamini-Hochberg corrected *P*-value ≤ 0.05 that were enriched for at least 2 given features.

**References**

Cahill KM, Huo Z, Tseng GC, Logan RW, Seney ML (2018) Improved identification of concordant and discordant gene expression signatures using an updated rank-rank hypergeometric overlap approach. Sci Rep 8**:**9588.

Castanza AS, Recla JM, Eby D, Thorvaldsdottir H, Bult CJ, Mesirov JP (2023) Extending support for mouse data in the Molecular Signatures Database (MSigDB). Nat Methods.

Chen EY, Tan CM, Kou Y, Duan Q, Wang Z, Meirelles GV, Clark NR, Ma'ayan A (2013) Enrichr: interactive and collaborative HTML5 gene list enrichment analysis tool. BMC Bioinformatics 14**:**128.

Csardi GNT (2006) The igraph software package for complex network research. InterJournal Complex Systems**:**1695.

Mitchell DC, Kuljanin M, Li J, Van Vranken JG, Bulloch N, Schweppe DK, Huttlin EL, Gygi SP (2023) A proteome-wide atlas of drug mechanism of action. Nat Biotechnol 41**:**845-857.

Ritchie ME, Phipson B, Wu D, Hu Y, Law CW, Shi W, Smyth GK (2015) limma powers differential expression analyses for RNA-sequencing and microarray studies. Nucleic Acids Res 43**:**e47.

Shannon P, Markiel A, Ozier O, Baliga NS, Wang JT, Ramage D, Amin N, Schwikowski B, Ideker T (2003) Cytoscape: a software environment for integrated models of biomolecular interaction networks. Genome Res 13**:**2498-2504.

Singh A, Shannon CP, Gautier B, Rohart F, Vacher M, Tebbutt SJ, Le Cao KA (2019) DIABLO: an integrative approach for identifying key molecular drivers from multi-omics assays. Bioinformatics 35**:**3055-3062.

Smyth GK (2004) Linear models and empirical bayes methods for assessing differential expression in microarray experiments. Stat Appl Genet Mol Biol 3**:**Article3.

Wu T, Hu E, Xu S, Chen M, Guo P, Dai Z, Feng T, Zhou L, Tang W, Zhan L, Fu X, Liu S, Bo X, Yu G (2021) clusterProfiler 4.0: A universal enrichment tool for interpreting omics data. Innovation (Camb) 2**:**100141.

Yoo M, Shin J, Kim J, Ryall KA, Lee K, Lee S, Jeon M, Kang J, Tan AC (2015) DSigDB: drug signatures database for gene set analysis. Bioinformatics 31**:**3069-3071.
