# Supplementary material for: Comparative multi‐omics in female mice reveals tissue‐specific vulnerabilities to chronic alcohol intake
Source: Alcohol Clin Exp Res (Hoboken). 2026 Jan 30;50(2):e70240. doi: 10.1111/acer.70240 (PMC12856532; doi:10.1111/acer.70240)
Supplement: Supplementary file 3 — Figure S1. [file ACER-50-0-s002.zip › Supplementary_legends_1.docx]

**Supplementary Information Files**

**Figure S1: Reactome Pathways enriched with genes dysregulated by chronic alcohol in the liver and/or muscle.** Bubble plot depicts results from over-representation analysis of MSigDB mouse Reactome Pathway gene sets for genes commonly/uniquely dysregulated by chronic alcohol use in the liver and muscle. Circle size is proportional to the number of gene hits as a % of the total number of annotated genes for a given overlap. Red and blue shading denote significant over-representation in upregulated and downregulated genes, respectively (adjusted *P* ≤ 0.05, enriched for ≥ 2 genes).

**Supplementary Document 1:** Omics based statistical analyses.

**Supplementary Excel 1:** File containing underlying values of reported data.
