# Supplementary figures and images for: Comparative multi‐omics in female mice reveals tissue‐specific vulnerabilities to chronic alcohol intake
Source: Alcohol Clin Exp Res (Hoboken). 2026 Jan 30;50(2):e70240. doi: 10.1111/acer.70240 (PMC12856532; doi:10.1111/acer.70240)

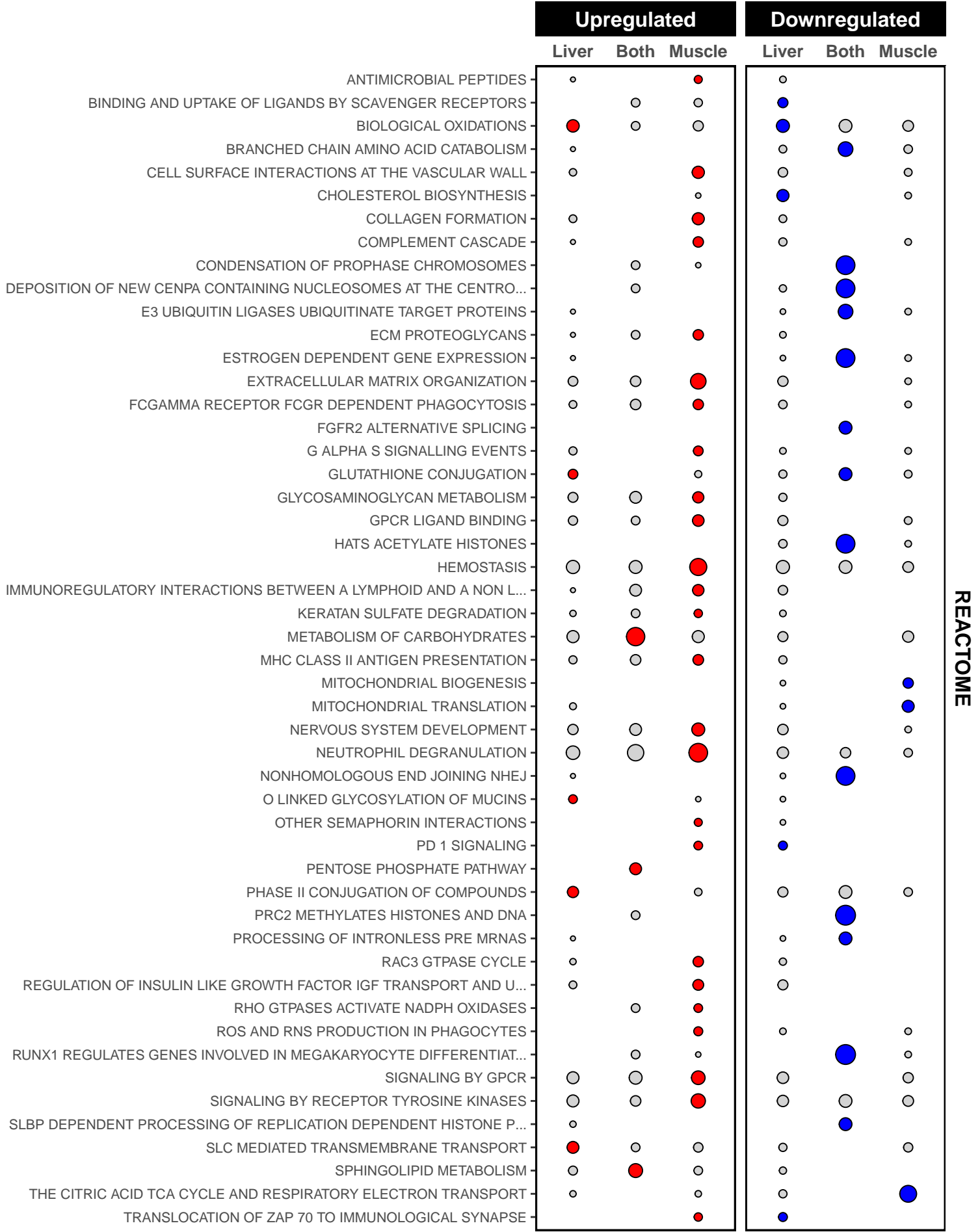

Supplement: Supplementary file 3 — Figure S1. [file ACER-50-0-s002.zip › acer70240-sup-0001-FigS1.pdf]
